# A Further Contribution to the Hygiene of Milk

**Published:** 1898-03

**Authors:** 

**Affiliations:** Switzerland


―――――――――――――――――――――――――――――
### THE JOURNAL OF COMPARATIVE MEDICINE AND VETERINARY ARCHIVES.

VOL. XIX. *MARCH, 1898.* No. 3.

### A FURTHER CONTRIBUTION TO THE HYGIENE OF MILK.

BY DR. OTT,
SWITZERLAND.

*Translated for "The Journal of Comparative Medicine" from the "Zeitschrift für Fleisch und Milch Hygiene," January, 1898.*

IN connection with an investigation in reference to the bacteria found in the local market milk, some samples were also examined for tubercle bacilli, and since they were determined microscopically in one case, a special series of experiments was undertaken with the view of making a study of this question. It has been shown frequently that tubercle bacilli are contained in milk that comes not only from cattle with tuberculosis of the udder, but also from cattle that suffer with tuberculosis elsewhere and even in a slight degree. A number of investigators have also shown that tubercle bacilli may occur in mixed milk as it comes to the market.

On account of the great importance of this question, and the fact that a large percentage of cattle suffer with tuberculosis, it seemed that it would be useful to make a special examination of market milk for the presence of tubercle bacilli. The investigation naturally covers two points: First, as to whether tubercle bacilli were actually present in the local market milk, and this must be determined by a microscopic examination; second, it must be determined whether the tubercle bacilli are virulent, and for this purpose animal experiments are necessary. In order to make a reasonably accurate microscopic examination of milk for tubercle bacilli it is necessary to centrifugalize it so that bacteria suspended in the milk can be gathered and examined microscopically in what might be called a concentrated form. But simple centrifugalizing of the milk will not suffice for this purpose, because not a few of the bacteria are carried to the surface by the fat-globules, and remain in the cream, and, therefore, do not appear in the sediment. Of the several methods that have been used to overcome this difficulty the author has used that recommended by Knut and Arnell to ascertain the presence of tubercle bacilli, who have employed a modification of the method of Röse-Gottlieb for determining the fat content.

The examination is carried out in the following manner: 25 c.c. of milk are placed in a mixing-cylinder that is pointed below and is provided there with a glass cock. To this, 2 c.c. of aqua ammonium are added. Then 100 c.c. of a mixture of equal parts of ether and petroleum-ether are added for the purpose of dissolving the fat, and the whole is thoroughly shaken. It is then allowed to stand until the watery and ethereal layers have completely separated. The watery ammoniacal solution of casein now contains all of the bacilli. This is drawn off by means of the glass cock and centrifugalized for fifteen minutes. In this way most of the bacteria are collected in the sediment. This method seems to furnish the simplest means of accomplishing this end. Samples of sediment are now fixed on cover-glasses in the usual way, stained with aniline-water-fuchsin, and treated further according to the method of Czaplewskis. In this way forty-three samples of milk were examined for tubercle bacilli. The milk was ordinary market milk, part of which had been submitted for a chemical examination and part of it was bought from dealers for this special purpose. The result of the investigation was as follows:

In the forty-three samples tubercle bacilli were found five times in the sediment obtained as above—that is, about 11.6 per cent. The number of tubercle bacilli was small in four cases, an occasional one being found only after examining several fields, while in one case a great many tubercle bacilli were present in every field. In this way the presence of tubercle bacilli in milk was proven.

Whether the tubercle bacilli were still virulent could only be determined by experiments on animals. For this purpose samples were purchased again from dealers in whose milk tubercle bacilli had been found, and guinea-pigs were inoculated with this material. In each case the milk was centrifugalized and 5 c.c. of a mixture of sediment and cream were injected. Further, the samples of milk were again examined for tubercle bacilli, according to the above method. The results of the experiments on animals were as follows:

*Sample of Milk No.* 1. Microscopically few bacilli; 5 c.c. were injected into the peritoneal cavity of two guinea-pigs. In twentytwo days one guinea-pig died, and the autopsy revealed a collection of light-yellow nodules on the peritoneum which, upon section, were found to be filled with a cheesy material. These were in the vicinity of the point of inoculation. On other portions of the parietal peritoneum were found small hard nodules. The omentum was contracted and studded with large and small grayish-white nodules. The folds of the intestine were grown to each other and to the abdominal walls. The spleen was enlarged and thickly studded with tubercles; lungs normal. Preparations made from the cheesy interior of the nodules of the peritoneum show a large number of tubercle bacilli.

The second animal was killed thirty days after injection, and autopsy revealed some small, hard, yellowish nodules around the point of inoculation. The spleen was enlarged and contained a few nodules the size of a pin-head. The retroperitoneal glands were swollen to the size of a pea. The liver, lungs, and kidneys were without macroscopic lesions. Preparations from the spleen and the nodules contained tubercle bacilli.

*Sample of Milk No.* 2. Microscpically no tubercle bacilli could be found at the second examination, although they were undoubtedly present at the previous examination. Both of the animals inoculated with this milk were killed in five weeks, and upon post-mortem examination were found to be free from tuberculosis.

*Sample of Milk No.* 3. About five tubercle bacilli were found in each field. Two guinea-pigs were injected intra-peritoneally, each with 5 c.c. of a mixture of sediment and cream. One was killed in twenty-eight days, and the other in thirty-five days after inoculation.

Autopsy: At the point of inoculation there was a large nodule almost entirely caseous. The peritoneum was thickly studded with nodules. Omentum was rolled up and lying in the greater curvature of the stomach, and contained *very large and small* nodules. The intestines adherent; the spleen enlarged, and upon cross-section was found to contain numerous caseous nodules. On the serous covering of the cæcum were two nodules the size of a pin-head. In the right kidney two large caverns connected with the pelvis of the kidney and contained numerous gray nodules. Lung tissue intact. In the diseased organs many tubercle bacilli were found.

*Sample of Milk No.* 4. Microscopically few bacilli. The animals were inoculated as before and killed in six weeks. By post-mortem examination one of them was found to contain nothing abnormal; while in the other there were several nodules at the point of inoculation and an enlarged spleen with nodules containing tubercle bacilli.

*Sample of Milk No.* 5. Microscopically few bacilli. One animal died forty days after inoculation, and the other was killed on the same day. In both animals there were lesions of tuberculosis. In the one that was killed the alterations were less extensive.

Therefore, in ten guinea-pigs inoculated with milk in which tubercle bacilli had been discovered microscopically, seven became infected with tuberculosis, and furnished proof that the tubercle bacilli were virulent. In reference to the sample of milk No. 2, in which tubercle bacilli were found originally, none could be found immediately before inoculation, and no infection was produced in the animals affected with it. The disappearance of the tubercle bacilli can be accounted for by the assumption that the contamination of the milk was from sputum or some other external cause.[1]

In connection with these experiments other guinea-pigs were inoculated, each with 5 c.c. of milk that was obtained from milk-dealers. This was injected into the peritoneal cavities under all of the necessary precautions. One animal died in thirty-six hours as a result of streptococcus infection; one died on the third day from peritonitis resulting from an injury to the intestine with the inoculating needle. The inoculations were made on January 4, 1897, and at the time of the preparation of this article[2] eight, in addition to the two above mentioned, have died. Of these eight animals four were undoubtedly infected with tuberculosis. In two of them the lesions were confined to the abdominal organs; in one there was a general miliary tuberculosis, and in the fourth the tubercles could not be regarded as the cause of death, for there were only small nodules on the peritoneum, some on the enlarged spleen and in a few glands. In this pig the kidneys, liver, omentum, and lungs were free from tuberculosis. The examination of the nodules on the peritoneum revealed the presence of tubercle bacilli, so that this case also must be regarded as one of mild infection. In reference to the other four that died, one showed a disease of the intestine, with bacteria in the blood that very closely resembled the colon-bacillus. The cause of death in the other three could not be discovered bacteriologically. Hence, if we disregard the two animals that died shortly after inoculation, of twenty-eight guinea pigs four

[1] It has been noted by several observers that tuberculous cows do not always furnish tuberculous milk every day.—[Translator.]

[2] Date not given.—[Translator.]

were infected with market milk—that is, 14 per cent. These results are modified by the fact that two samples of milk which produced tuberculous infection came from the same herd, being sold by different dealers, hence only three samples of milk can be regarded as containing tubercle bacilli, and the percentage is reduced to 10.7 per cent.

The fact that milk as it comes to the market may contain virulent tubercle bacilli is of the greatest hygienic significance. While it is possible by sterilization to kill the tubercle bacilli that milk may contain, we must remember that a large proportion of milk is consumed in a raw state, indeed, that cow-warm milk is used as a medicine in a number of diseases, as anæmia, debility, etc. We must also remember that the palatability of milk is lessened by cooking, and for this reason the fresh, uncooked milk is preferred, so that the hygienic recommendation to cook all milk before it is used is usually disregarded, except in the feeding of children. But even here, and generally in the "sterilizing" of milk by the laity, sterilization is frequently not accomplished, as is shown by an example of Legay who found virulent tubercle bacilli in milk that was supposed to have been sterilized by heat. These conditions are not very favorable to the public health, and especially when the further point is considered that not only the milk, but also its products, especially the butter, can convey the infection. Attention has already been called to the fact that when milk is allowed to stand or is centrifugalized the tubercle bacilli or their microörganisms are carried upward by the fat-globules, and so enter the cream and the butter. Reports of butter containing tubercle bacilli have been frequent. Among others by Roth, Brusaferro, Obemüller, and others. It must be admitted that, with us, the public, and especially the lower classes, have a prejudice against the sterilization of milk. The knowledge of bacteria and their work has scarcely been disseminated among such classes at all, excepting in caricatures and jokes.[1]

As long as the public is not informed as to the various possibilities of infection, and so long as it is not convinced of the transmission of the diseases of animals to man, little improvement can be expected in the field of milk-hygiene in spite of the boards of health, district veterinarians, and compulsory notification. Improvement is obstructed by the fact that the examination of milk for tubercle bacilli is neither simple nor rapid. If we had a rapid and accurate method of determining tubercle bacilli in milk, the suspected samples could be set aside and kept out of the market.

[1] Also true in this country!—[Translator.]

Unfortunately, no such method exists. It is true that a number of signs have been given whereby one is supposed to be able to detect the milk of tuberculous cattle. It is said that it forms cream rapidly, that the cream and milk are prone to undergo putrefaction, and that the milk does not clot. But the value of all these signs is problematic, because they only occur when the milk comes from animals that are extensively tuberculous, and not in mixed milk or milk from cows that are slightly tuberculous.

The ideal and desirable condition in reference to the danger of tuberculous infection from milk would be reached if no milk from tuberculous or suspected animals were sold, especially in view of the experiments of de Michele, who has found toxins in the milk of tuberculous cows, so that it is not only the tubercle bacilli in such milk, but also the poisonous metabolic products that are injurious to the consumer. However desirable as this goal is, it will, nevertheless, be very difficult to reach it in practice. We are not in a position to ascertain the existence of tuberculosis in every case with absolute accuracy, especially in the beginning stages, so we must seek means of lessening the possibility of the milk of tuberculous cows getting on the market, and of destroying the virulence of whatever bacteria milk may contain. For the second object we have sterilization, and this can be done on a large scale in central institutions, or on a small scale in the individual families. Of this something will be said in another connection. It is of more interest here to consider the regulations by which the sale of milk containing tubercle bacilli may be prevented.

As a first measure in this direction, it is necessary to inform the milk-producers as to the possibilities and dangers of the transmission of tuberculosis and other diseases to people through the consumption of milk from diseased cattle. This instruction should also be directed to the end that owners of cattle shall more fully recognize that it is necessary to have a careful veterinary control of milk-cows, and that they must be examined as to their health from time to time, so that the milk of diseased animals can be kept out of the market, all of this being necessary in order to meet the requirements of modern hygiene. In this way the most important service could be rendered to the subject of milk-hygiene. Above all, it is necessary that milk-sanitaria (*milch kuranstalten*) and dairies conducted for similar purposes should be under a careful veterinary supervision. Unfortunately, this inspection is not often required by us, and Vogel has justly said: "A stable that is half clean, and a herd of Swiss cattle that are fed on dry feed, meet the popular requirement as to a complete dairy, and when it has the recommendation of a physician, it receives the name of milk-sanitarium; it supplies milk for children and infants, and a double price is charged for its product. Whether the cows are healthy or well-fed is not a matter that the public often troubles itself about."

While a permanent veterinary inspection is urgently demanded in reference to the retail milk-producers and the milk-sanitaria, the eventual extension of this inspection to all milk-producers is not to be disregarded. While the milk-control that is usually carried out gives information as to the addition of water, the removal of fat, and in some cases as to the presence of dirt in milk, the matter of freedom from pathogenic organisms or the health of the cows supplying the milk is not covered, although this last requirement is of far greater hygienic importance than the first. It is undoubted that tuberculous cows furnish milk containing tubercle bacilli, and as our knowledge of hygiene is opposed to the sale and use of such milk, it is necessary to grasp this evil at its root and exclude diseased cows from dairies producing market milk.

The diagnosis of disease and the development of appropriate regulations to this end is a matter for experienced veterinarians, but everything indicates that in tuberculin we have a diagnostic agent that makes it possible to recognize even concealed tuberculous infections. But the veterinarian should not confine his attention entirely to the health of the animals; he should also consider the methods followed in the dairy, the feeding, the bedding, the cleanliness, etc.

As to the examination of milk for tubercle bacilli, this is of great importance in connection with milk-control, especially in cities. It serves for the extension and support of the other regulations. To make the entire control depend upon this one point is impossible, for the negative result of an examination does not furnish us with sufficient evidence as to the health of the animal furnishing the milk. The first requirement for the removal of the dangers that accompany the use of milk from diseased animals is systematic veterinary examination and control of all animals that produce milk for human consumption, and the exclusion from the dairy of every diseased or in any way suspicious animal. There are at present so many ways and means that are being considered for combating infectious diseases that are demanding public assistance that one is justified in expressing a wish that the Government should direct its attention to the dangers of infection through the consumption of milk and to the development of the hygiene of this subject, to the end that there may be a public control of the entire milk-supply from its origin to the consumer. This control and its results would be very difficult in the beginning, but it would be the means of saving many children and would remove a great many evils whose existence and effects will hardly be appreciated until they cease to exist.

